# Aquatic eDNA for monitoring French Guiana biodiversity

**DOI:** 10.3897/BDJ.7.e37518

**Published:** 2019-09-11

**Authors:** Jérôme Murienne, Isabel Cantera, Axel Cerdan, Kévin Cilleros, Jean-Baptiste Decotte, Tony Dejean, Régis Vigouroux, Sébastien Brosse

**Affiliations:** 1 Laboratoire Evolution et Diversité Biologique (EDB UMR5174) CNRS, Université Paul Sabatier Toulouse 3, IRD, Toulouse, France Laboratoire Evolution et Diversité Biologique (EDB UMR5174) CNRS, Université Paul Sabatier Toulouse 3, IRD Toulouse France; 2 VigiLIFE 17, rue du Lac Saint-André Savoie Technolac - BP 10366, Le Bourget du Lac, France VigiLIFE 17, rue du Lac Saint-André Savoie Technolac - BP 10366 Le Bourget du Lac France; 3 SPYGEN, 17 rue du Lac Saint-André Savoie Technolac - BP 274, Le Bourget du Lac, France SPYGEN, 17 rue du Lac Saint-André Savoie Technolac - BP 274 Le Bourget du Lac France; 4 HYDRECO, Laboratoire Environnement de Petit Saut, B.P 823, F-97388, Kourou, French Guyana HYDRECO, Laboratoire Environnement de Petit Saut, B.P 823, F-97388 Kourou French Guyana

**Keywords:** South America, French Guiana, metabarcoding, environmental genomics

## Abstract

**Background:**

Environmental DNA [eDNA] metabarcoding has recently emerged as a non-destructive alternative to traditional sampling for characterising species assemblages.

**New information:**

We here provide a consistent dataset synthetising all eDNA sampling sites in French Guiana to date. Field collections have been initiated in 2014 and have continued until 2019. This dataset is however a work in progress and will be updated after each collecting campaign. We also provide a taxon by site matrix for fishes presence / absence as inferred from eDNA. Our aim is to allow a transparent communication to the stakeholders and provide the foundation for a monitoring programme based on eDNA. The lastest version of the dataset is publicly and freely accessible through the CEBA geoportal (http://vmcebagn-dev.ird.fr) or through the French Guiana geographic portal (https://www.geoguyane.fr).

## Introduction

French Guiana is an overseas territory of France located on the north-eastern coast of South America. With ca. 84,000 km (the size of Austria), it represents the largest outermost region of Europe. About 96% of its surface is covered by undisturbed primary rainforest. Due to its location in a tropical humid environment, the territory harbours a very dense hydrographic network. This network is comprised of 112,000 km of water bodies and is divided into 8 drainage basins flowing south-north (Mourguiart and Linares 2013). As opposed to Amazonia *sensu stricto*, where all the basins are connected to the Amazon, French Guiana basins are all disconnected and independently lead to the Atlantic Ocean. The two largest basins, the Maroni and the Oyapock, are boundaries with Suriname and Brazil, respectively. A total of 20% of the network is represented by rivers (Strahler order > 3) while the remaining 80% correspond to streams less than 10 m large and less than 1 metre deep.

As a European territory, French Guiana must comply with European regulations aiming at developing surveillance programmes on water quality (Directive 2000/60/EC). This directive was translated into French law (n°2004-338) mainly under article R212-22 of the environment code and the “Law on water and aquatic environment” (n°2006-1772). For the territory of French Guiana, several surveillance programmes have been set up for the time periods 2010-2015 and 2016-2021. This has resulted in a characterisation of both reference physico-chemical environments and biological communities, as well as practical tools (e.g. biological indices) to evaluate and monitor water quality. A set of sites have been defined under the “Surveillance Control Network” and the “Operational Control Network” that are monitored on a yearly basis.

However, quantifying the composition of species assemblages in Amazonian aquatic systems remains difficult because species inventories are harmful to the fauna. Indeed, sampling fish in small streams consists in the use of toxicant (rotenone) that kill all the fishes within the stream reach (Allard et al. 2014). In rivers, gill nets are used and cause lethal injuries to the fishes entangled in the nets (Murphy and Willis 1996). Such destructive sampling no longer complies with ethics and European laws. Non-destructive methods, such as diving and electrofishing are not efficient in those streams and rivers due to their low water conductivity and their high turbidity (Allard et al. 2014, Melki 2016). As a consequence, collecting data on entire assemblages is almost impossible using traditional sampling methods, which act as a barrier to scientific advances on ecosystem structure and function and associated applied issues on biodiversity conservation and management.

Since 2014, we used a non-destructive alternative to traditional fish sampling by characterising species assemblages using environmental DNA [hereafter eDNA] metabarcoding (Taberlet et al. 2018, Taberlet et al. 2012). eDNA consists of collecting DNA released by organisms directly into the water. Environmental DNA sequences are then compared to reference molecular databases to assign sequences to species. This method has been shown to efficiently characterise fish faunas in temperate rivers (Civade et al. 2016, Valentini et al. 2016) and has recently been successfully applied in French Guiana (Cilleros et al. 2019, Cantera et al. 2019). We here provide a consistent dataset synthetising all eDNA sampling sites in French Guiana to date. We also provide a taxon by site presence/absence matrix for the fish fauna. Our aim is to allow a transparent communication to the stakeholders and provide the foundation for a monitoring programme based on eDNA.

## Project description

### Title

Aquatic eDNA samples in French Guiana

### Personnel

Personnel involved in data aquisition (by alphabetic order): Sébastien Brosse, Isabel Cantera, Axel Cerdan, Kévin Cilleros, Jean-Baptiste Decotte, Gaël Grenouillet, Amaia Iribar, Jérôme Murienne, Pierre Taberlet, Pablo Tedesco and Régis Vigouroux.

### Study area description

Collecting trips have been conducted in various locations throughout French Guiana.

### Design description

This dataset was developed to provide the foundation for a biodiversity monitoring programme based on eDNA but also to better understand the impact of human activities on aquatic biodiversity. Locations were thus selected to maximise the geographic coverage of rivers and streams, taking into account undisturbed sites but also sites under human disturbances (close to villages, close to gold mining sites etc.).

### Funding

Data for this resource have been obtained with support from Labex CEBA (Center for the Study of Biodiversity in Amazonia), Labex DRIIHM (Dispositif de Recherche Interdisciplinaire sur les Interactions Hommes-Milieux) and Labex TULIP (Towards a Unified theory of biotic interactions: role of environmental perturbations). Labex (Laboratoires d’Excellence) are funded by "Investissement d'Avenir" grants managed by the French National Research Agency (ANR) under references ANR-10-LABX-25-CEBA, ANR-11-LABX-0010-DRIIHM and ANR-10-LABX-0041-TULIP. Additional financial support was also obtained from the DEAL Guyane, Office de l’Eau Guyane (Aquatic Metabarcoding project) and through the ANR DEBIT project (ANR-17-CE02-0007-01). SPYGEN, a private company specialised in eDNA, as well as VigiLife, a non-governmental agency, provided financial and laboratory support. Logistic support was also provided by the Parc Amazonien de Guyane and Hydreco Laboratory (Kourou, Guyane).

## Sampling methods

### Study extent

**Figure 1.**
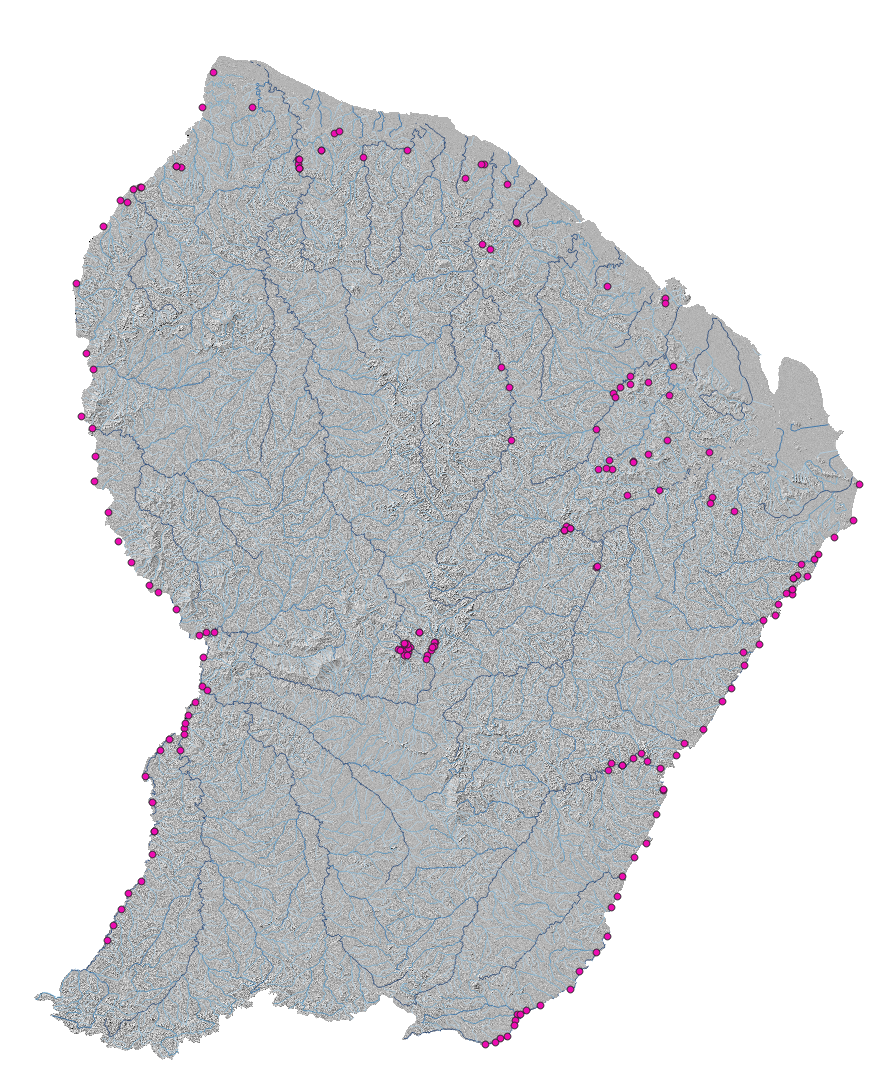
Localisation of the environmental DNA sampling sites.

Sampling sites were located throughout French Guiana Fig. 1.

### Sampling description

We collected eDNA samples from November 2014 to 2019. For sampling, laboratory and bioinformatic protocols, we followed Valentini et al. (2016) from 2014 to 2016 and Pont et al. (2018) since 2016. For each sample, we used a filtration kit made of a sterile, single use filtration cartridge (Enviroteck HV; Pall Corporation, Ann Arbor, MI, USA and VigiDNA 0.45 μm; SPYGEN, le Bourget du Lac, France), a peristaltic pump (Vampir Sampler; Bürkle GmbH, Bad Bellingen, Germany) and sterile, single-use tubing. All the materials were handled with sterile gloves. Initial sampling (2014-2015) was performed using a 1 micrometre filtration cartridge (Enviroteck HV; Pall Corporation, Ann Arbor, MI, USA) but 0.45 micron capsules (VigiDNA 0.45 μm; SPYGEN, le Bourget du Lac, France) have been used as standard since 2016. Most of the samples consisted of 30 minutes water filtration using a portable battery powered peristaltic pump (Vampir sampler, Burkle, Germany), but in a few sites, filtration time was reduced to 15 minutes. A single sample per site was collected during initial sampling (2014-2015). Cantera et al. (2019) collected 10 replicate samples in 6 selected sites and showed that two replicate samples per site provided a realistic species list while limiting sampling costs. Two replicate samples were therefore collected in each site since 2016.

### Quality control

The operator always remained downstream from the filtration area and stayed on the bank (for small streams) or on emergent rocks (for larger streams and rivers). For sites located along the same river course, we sampled downstream to upstream to avoid contamination by eDNA transported by the boat (for rivers) or clothes.

Geographical coordinates were obtained using a GPSmap 64S device (Garmin) or similar. Such devices report coordinates accuracy using the CEP50 (Circular Error Probability), meaning that there is only 50% probability that a reported position would be within a distance of X metres to the real position. Considering other sources of GPS errors (such as ionosphere delay and signal multi-path), we estimate the accuracy of the coordinates to be around 30 metres at a 95% confidence level under dense forest cover.

### Step description

At each site, we placed the input part of the tubing in a high-flow part of the watercourse. Sampling was achieved in rapid hydromorphological units to ensure an optimal homogenisation of the water throughout the water column. Water was pumped ca. 20 cm below the surface and each filtration lasted 30 min (except for a few sites where filtration time was 15 minutes). Each sample results from the filtration of ~34 l of water (~17 litres when filtration time was 15 minutes). At the end of the filtration, we emptied the filtration capsule of water, filled it with 150 ml of preservation buffer (Tris–HCl 0.1 M, EDTA 0.1 M, NaCl 0.01 M and N-lauroyl sarcosine 1%, pH 7.5–8) and stored it in the dark in individual sterile plastic bags. Samples were then stored at room temperature before DNA extraction. Preliminary tests demonstrated that the preservation buffer was suitable for room temperature storage up to a month. Information on DNA extraction, amplification and sequencing, as well as subsequent bioinformatic pipelines, can be found in Cilleros et al. (2019) and Cantera et al. (2019).

Site scale variables were measured directly in the field at the sampling location. Width was measured using a decameter for small streams (less than 15 metres width and 1 metre depth) and using an electronic telemeter (Bushnell Sport 850) for larger rivers. Water depth was measured using a graduated stick in small streams and a depth sounder (Plastimo echotest II) in larger rivers. Turbidity was measured using a Eutech Instrument Turbimeter (TN-100). Temperature, O_2_ saturation, O_2_ and pH were measured using a WTW 3420 field multimeter. Geographical coordinates were obtained using a GPSmap 64S device (Garmin) or similar. Elevation was derived for the geographic coordinates using the SRTM30 dataset.

## Geographic coverage

### Description

The sampling area is delimited by the current administrative boundaries of the French Guiana territory. To the East, the Oyapock river delimits the frontier with Brazil. To the West, the Maroni river delimits the frontier with Suriname. This is an important detail as the delimitation of the territory has not been constant throughout history and a large portion of northern Brazil was disputed between France and Brazil during the 19th century. Even though French Guiana is an overseas territory of France, all occurrences are considered as belonging to the French Guiana "country" to comply with the ISO 3166-1 standard.

### Coordinates

2.00000 and 6.00000 Latitude; -51.5000 and -54.5000 Longitude.

## Taxonomic coverage

### Description

The dataset provides information on eDNA sampling sites and fishes presence/absence as inferred from metabarcoding analyses (Cilleros et al. 2019). DNA extracted from the sampling cartridge could, in theory, be used for amplifying any taxonomic group, depending on the downstream molecular biology protocols. Local metabarcoding reference databases for French Guiana biodiversity are currently available for mammals (Kocher et al. 2017b, Kocher et al. 2017a) and insects (Talaga et al. 2017, Kocher et al. 2016), but additional databases are under active development for other groups as well.

## Temporal coverage

### Notes

2014-2019

## Usage rights

### Use license

Creative Commons Public Domain Waiver (CC-Zero)

### IP rights notes

Users of this resource should comply with the CEBA data sharing agreement available here: www.labex-ceba.fr/assets/CEBA_Data_Sharing_Agreement_nov2013.pdf

## Data resources

### Data package title

Aquatic eDNA for monitoring French Guiana biodiversity

### Resource link

http://vmcebagn-dev.ird.fr/geonetwork/srv/eng/search?=eng#|5617a9ff-d0aa-48a9-b2c2-cb7fd5b92692

### Alternative identifiers

5617a9ff-d0aa-48a9-b2c2-cb7fd5b92692

### Number of data sets

2

### Data set 1.

#### Data set name

Aquatic_eDNA_[date]

#### Data format

ESRI Shapefile (a spreadsheet in "tab separated value" format is also provided for compatibility).

#### Number of columns

23

#### Description

This dataset provides detailed information on sampling sites and sampling events. The latest version of the dataset is available on the CEBA geoportal (http://vmcebagn-dev.ird.fr) under reference 5617a9ff-d0aa-48a9-b2c2-cb7fd5b92692.

**Data set 1.** 

Column label	Column description
Site code	A unique identifier of the site that could be used for downstream analyses (optional).
Site name	The name of the sampling location.
Site description	The original textual description of the site.
Drainage Basin	The name of the drainage basin (either Oyapock, Aprouague, Comte, Sinamary, Organabo, Iracoubo, Mana, Maroni).
Latitude	The geographic Latitude (in decimal degrees, WGS84) of the sampling point.
Longitude	The geographic Longitude (in decimal degrees, WGS84) of the sampling point.
Elevation	Altitude in metres above sea level inferred from the geographic coordinates and the SRTM30 dataset.
Watercourse class	The watercourse class infered a posterio based on the BD Carthage dataset.
Event date	The date of the sampling event.
Disturbance	Level of disturbance at the site (either Reference for undisturbed site, gold mining, ancient gold mining, agriculture and/or urbanisation). Estimated a priori.
Depth	Water depth in metres (measured at the sampling site).
Width	Watercourse width (in metres) measured at the sampling site.
Conductivity	Water conductivity (in micro Siemens) measured at the sampling site using a WTW 3420 field Multiparameter fitted with a TetraCon 925 conductivity probe
Temperature	Water temperature (in degree Celcius) measured at the sampling site.
pH	Water pH measured at the sampling site using a WTW 3420 field Multiparameter fitted with a SenTix 940-3 pH probe.
Turbidity	Turbidity (in NTU) measured at the sampling site by a EUTECH TN-100 field turbidimeter.
O_2_	O_2_ (in milligram per litre) measured at the sampling site using a WTW 3420 field Multiparameter fitted with a FDO925 Oxygen probe.
0_2_ saturation	O_2_ saturation (in percent) measured at the sampling site using a WTW 3420 field Multiparameter fitted with a FDO925 Oxygen probe.
Salinity	Water salinity measured at the sampling site using a WTW 3420 field Multiparameter
Time	Filtering time (in minutes)
Filter	Filter size (in micrometres)
Nb_replicates	Number of replicates
replicatX	For each replicate, the unique filter identifier

### Data set 2.

#### Data set name

Aquatic_eDNA_fishData_[date]

#### Data format

Spreadsheet in "tab separated value"

#### Number of columns

1

#### Description

This dataset provides a taxon by site matrix, made after sequences assignment to the reference database (Cilleros et al. 2019). For taxa described at the genus level or higher, the number of included species is indicated within parentheses. The latest version of the dataset is available on the CEBA geoportal (http://vmcebagn-dev.ird.fr) under reference 5617a9ff-d0aa-48a9-b2c2-cb7fd5b92692.
